# Advanced hybrid closed loop system (MiniMed 780G) achieving significant improvement of glucose control in a patient with maternally inherited diabetes and deafness: a case report

**DOI:** 10.1007/s00592-025-02524-0

**Published:** 2025-06-05

**Authors:** Michalis K. Picolos, Anastasia Papapostolou, Anastasia Christodoulou, George A. Tanteles, Anna Kyriakidou, Georgios Papaetis

**Affiliations:** 1 Alithias Endocrinology Centre, 9A Alithias Street, Kato Lakatamia, Nicosia, 2324 Cyprus; 2 European University, Nicosia, Cyprus; 3 Nikis Endocrinology Outpatient Clinic, Nicosia, Cyprus; 4 Department of Basic and Clinical Sciences, University of Nicosia Medical School, Nicosia, Cyprus; 5 Clinical Genetics and Genomics Department, The Cyprus Institute of Neurology and Genetics, Nicosia, Cyprus; 6 Barnet Hospital, Royal Free London NHS Foundation Trust, London, UK; 7 Internal Medicine and Diabetes Clinic, K.M.P THERAPIS Paphos Medical Center, Paphos, Cyprus; 8 CDA College, 73 Democratias Avenue, Paphos, Cyprus

**Keywords:** Mitochondrial diabetes, Maternally inherited diabetes and deafness, MIDD, Insulin pump, AID, Advanced hybrid closed loop, AHCL, MiniMed 780G, Medtronic.

Maternally inherited diabetes and deafness (MIDD) or mitochondrial diabetes is a multi-system disorder that follows matrilineal inheritance caused by pathogenic variants in the mitochondrial DNA (mtDNA) thus leading to mitochondrial dysfunction. The clinical picture can be variable and mainly depends on the degree of admixture between wild type and mutated mtDNA alleles in an individual (heteroplasmy). It is believed that some patients may be asymptomatic if they do not meet the heteroplasmic threshold, whereas others may exhibit atypical diabetes with progressive symmetrical sensorineural hearing impairment or additional manifestations from the brain, heart, kidneys, retina, peripheral muscles or skeletal nerves [1, 2]. The most common pathogenic variant identified is the A to G transition at position 3243 within the *MT-TL1* gene encoding the mitochondrial tRNA^Leu(UUR)^, [NC_012920.1(MT-TL1):m.3243 A > G]. Diabetes in MIDD is believed to be caused by a gradual decrease in insulin production due to reduced manufacturing of ATP by the pancreatic cells with mutated mitochondria and by increased oxidative stress from reactive oxygen species [3, 4]. The prevalence of MIDD amongst patients with type 2 diabetes is believed to be about 1% with most reported being mainly of Asian and secondly of European descend. The mean age of diabetes onset is in the early thirties but can develop at any age [1, 3].

Our patient is a 54-year-old lean Cypriot female [body mass index (BMI) 21.8 kg/m^2^] with mitochondrial diabetes, harboring the *MT– TL1* 3243 A > G pathogenic variant, hypoglycemia unawareness, sensorineural hearing impairment, myopathy, polyneuropathy, hyperlipidemia, hypertension, surgical hypothyroidism due to stage 1 papillary thyroid cancer, post-thyroidectomy hypoparathyroidism, non-functioning pituitary microadenoma, hypovitaminosis D, gastritis, dolichocolon, and anxiety. She presented to our center with a request to initiate Advanced Hybrid Closed Loop (AHCL) system. She had no known retinopathy, nephropathy, peripheral vascular disease or coronary artery disease.

Her medications included basal/bolus insulin with Degludec 36 units every morning and Novorapid with carbohydrate counting prior to each meal (1 unit for every 6 g of carbohydrates and an additional unit for every 30 mg/dL of blood glucose above 100 mg/dL), Ezetimibe, Rosuvastatin, Candesartan, Bisoprolol, Levothyroxine, Calcium carbonate, One Alpha, Coenzyme Q10 and multiple vitamins.

Diabetes was diagnosed at age 29 and was initially treated with oral hypoglycemics (metformin and sulfonylureas) but about 2.5 years later she was started on insulin due to insufficient glucose control. Initially she was administering Protaphane HM at night with Actrapid HM in fixed doses for meals and three years later Glargine daily with fixed doses of Novorapid for meals and additional units for corrections. Despite these changes her diabetes control had been deteriorating. Additionally, in the last five years she started developing hypoglycemia unawareness. One year prior to the presentation to our center she had an episode of severe hypoglycemia with loss of consciousness. Following that episode, her basal insulin was switched to Degludec and the bolus regimen (Novorapid) was calculated with carbohydrate counting and correction doses after appropriate education. Continuous Glucose Monitor [(CGM) Medtrum Touchcare S9 system] was also added over the last six months prior to her presentation. *U*nfortunately, she continued to experience significant glucose variability with episodes of hyperglycemia and hypoglycemia (Fig. 1A). Her glycated hemoglobin (HbA1c) over the last 3 years had been ranging from 6.5 to 8.5% with most being above 7%. Her C-peptide upon presentation to our center was 0.899 ng/mL (normal 1.1–4.4) with blood glucose of 120 mg/dL and HbA1c was 7.3%. After the episode of severe hypoglycemia, her anxiety and fear of hypoglycemia intensified causing worsening of insomnia and affecting her quality of life so she decided to seek the assistance of an AHCL system in order to improve both these parameters as well as her glucose control.

**Fig. 1:**
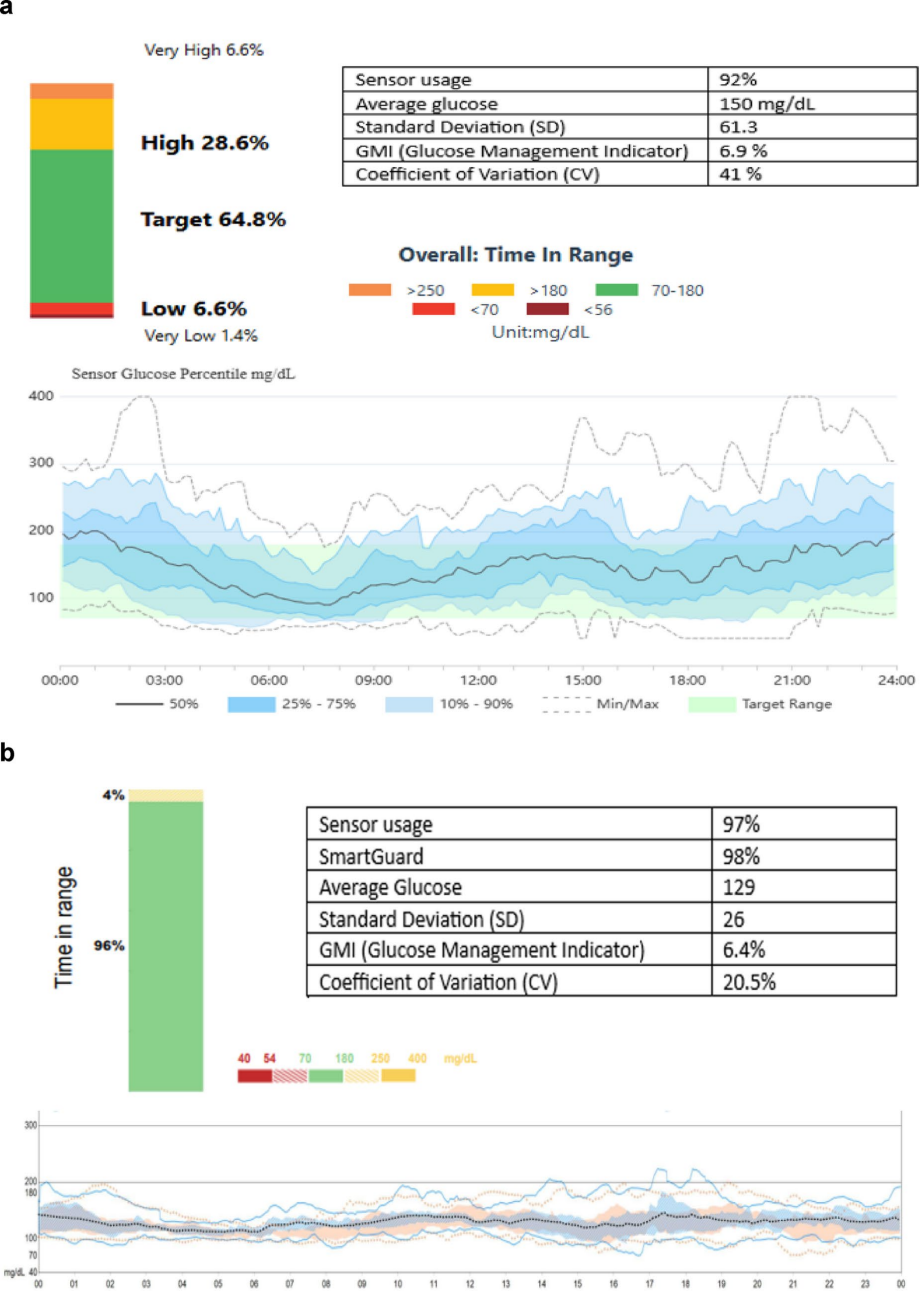
Continuous glucose monitoring data prior and after AHCL system initiation. **a** Medtrum Touchcare S9 system data for 2 weeks (prior to MiniMed *780G* initiation). **b** Medtronic Guardian 4 sensor data for 2 weeks (4 months after MiniMed780 initiation)

Off label AHCL therapy with MiniMed 780G system (MM780G) was initiated after appropriate training, about one month following the initial presentation to our center, with improvement of glycemic control (Fig. 1B). Her time in range (TIR), time below range (TBR) < 70 mg/dL and coefficient of variation (CV) prior to AHCL system initiation were 64.8%, 8% and 41% respectively and improved to 96%, 0%, 20.5% four months after the system initiation (Fig. 1). These changes had been evident even from the first days of AHCL system introduction. Four months *after AHCL* the patient’s weight dropped by 3 kg (BMI 20.3 Kg/m2) due to the significant amelioration of carbohydrate consumption to correct or avoid hypoglycemia, total insulin dose decreased from about 55 units/day to 39 units/day, and HbA1c was down to 6.3%. Additionally, she expressed decreased anxiety, better sleep quality, decreased fear of hypoglycemia and improved quality of life.

Management of MIDD includes mainly supplementation with CoQ10 which is an antioxidant and mitochondrial cofactor that may enhance insulin secretion, slow hearing loss, improve symptoms of myopathy and congestive heart failure [5, 6]. Medications that pose the risk of mitochondrial toxicity can be used with caution. *Diabetes* treatment includes diet only, in a minimal proportion of MIDD patients. Oral hypoglycemics and novel non-insulin therapies have been used [7]. Metformin should be avoided due to the risk of lactic acidosis. A significant proportion of patients however, like our own, will eventually need insulin due to the decline in beta cell function. In a recent study of 161 patients with MIDD 77.45% were using insulin with mean time from diabetes diagnosis to insulin initiation of 4.15 years [1]. To our knowledge the use of an *automatic insulin delivery (AID) or AHCL* system has not been reported in patients with MIDD.

Diabetes technologies have revolutionized the treatment of type 1 diabetes. AHCL therapy, one of the latest additions in our therapeutic armamentarium has demonstrated higher TIR, equal or less hypoglycemia and decreased HbA1c. It combines a CGM with an *insulin pump* and an algorithm to automatically adjust insulin delivery (AID - Hybrid Closed loop system) with additional features. Manual boluses for meals are required. MM780G is an AHCL system which integrates as additional function of automated correctional insulin boluses to address hyperglycemia as needed up to every 5 min. The safety and efficacy of MM780G have been reported in clinical as well as in real world studies [8].

AID and AHCL systems have also been successfully used in type 2 diabetes (T2D), special situations and rare forms of diabetes [9–14]. They seem to significantly increase TIR, decrease average glucose and glycemic variability without significantly altering TBR. In a recent study of 95 adults with T2D, the use of MM780G for 90 days decreased in a statistically significant way HbA1c by 0.7% (from 7.9% to 7.2%) and increased TIR by 7.6% (from 72.2% to 79.8%) [15]. Changes in TBR and CV did not reach statistical significance. In a pilot study of 10 patients with cystic fibrosis the use of AHCL system for one year (Tandem t: slim X2 Control IQ or MM780G) was able to improve in a statistically significant way HbA1c by 0.96% (reduction from 7.31% to 6.35%), TIR by 16.17% (increase from 60% to 76.17%) and CV by 8.77% (reduction from 39% to 30.23%) [16]. TBR change did not reach statistical significance. In our patient with MIDD, MM780G was able to attain impressive improvement of (i) TIR increasing it by 31.2% to reach 96%, (ii) TBR decreasing it by 8% to reach 0% and (iii) CV decreasing it by half to reach 20.5%. HbA1c was also reduced by 1% to 6.3%. The above achievements were also translated into lessened fear of hypoglycemia and better quality of life. These results suggest that ACHL systems should be considered as a valid therapeutic option in patients with MIDD on insulin treatment who do not meet their glycemic targets. Additionally, AHCL systems could be a potential alternative for all patients with MIDD on insulin therapy. Data on the cost effectiveness, long-term glycemic benefits and health-related quality of life of this approach however are lacking and further studies are needed to clarify these important issues.

## Data Availability

Original data generated and analyzed during this study are included in this published article and are available from the corresponding author upon request.
